# In Silico Screening of Natural Compounds for Candidates 5HT6 Receptor Antagonists against Alzheimer’s Disease

**DOI:** 10.3390/molecules27092626

**Published:** 2022-04-19

**Authors:** Tijana Bojić, Milan Sencanski, Vladimir Perovic, Jelena Milicevic, Sanja Glisic

**Affiliations:** 1 Laboratory of Radiobiology and Molecular Genetics-080, Institute of Nuclear Sciences Vinca, National Institute of the Republic of Serbia, University of Belgrade, P.O. Box 522, 11000 Belgrade, Serbia; 2 Laboratory for Bioinformatics and Computational Chemistry, Institute of Nuclear Sciences Vinca, National Institute of the Republic of Serbia, University of Belgrade, P.O. Box 522, 11000 Belgrade, Serbia; vladaper@vin.bg.ac.rs (V.P.); jdjordjevic@vin.bg.ac.rs (J.M.); sanja@vinca.rs (S.G.)

**Keywords:** molecular docking, ligand-based virtual screening, ADMET calculations, FEP simulations

## Abstract

Alzheimer’s disease (AD), a devastating neurodegenerative disease, is the focus of pharmacological research. One of the targets that attract the most attention for the potential therapy of AD is the serotonin 5HT6 receptor, which is the receptor situated exclusively in CNS on glutamatergic and GABAergic neurons. The neurochemical impact of this receptor supports the hypothesis about its role in cognitive, learning, and memory systems, which are of critical importance for AD. Natural products are a promising source of novel bioactive compounds with potential therapeutic potential as a 5HT6 receptor antagonist in the treatment of AD dementia. The ZINC—natural product database was in silico screened in order to find the candidate antagonists of 5-HT6 receptor against AD. A virtual screening protocol that includes both short-and long-range interactions between interacting molecules was employed. First, the EIIP/AQVN filter was applied for in silico screening of the ZINC database followed by 3D QSAR and molecular docking. Ten best candidate compounds were selected from the ZINC Natural Product database as potential 5HT6 Receptor antagonists and were proposed for further evaluation. The best candidate was evaluated by molecular dynamics simulations and free energy calculations.

## 1. Introduction

**Figure 1:**
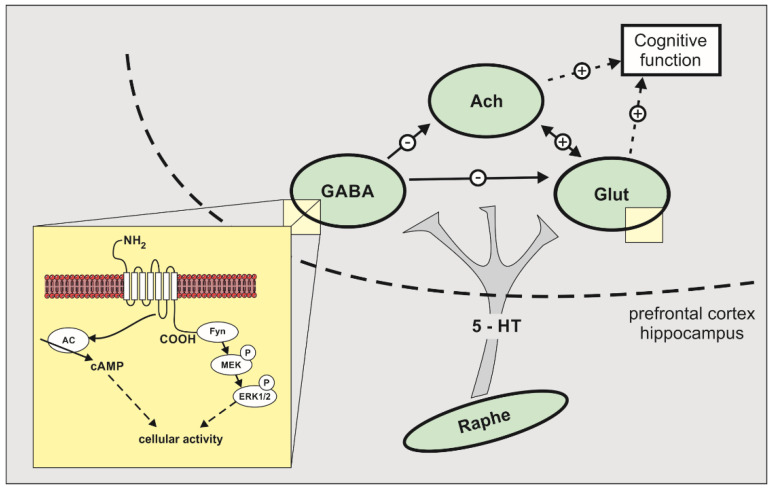
Neurochemical and biochemical mechanisms mediating 5-HT6 receptor functions. In addition to the activation of cAMP signalling pathways, 5-HT6 receptors activate the extracellular signal regulated kinase1/2 (ERK1/2) via Fyn-dependent pathway. The proposed neurochemical circuitry for 5-HT6 receptors to influence cognition involves modulation of cholinergic and/or glutamatergic activity through GABAergic interneurons. AC: adenylate cyclase; ACH: acetylcholine; Glut: glutamate. (+): stimulation; (—): inhibition. (adapted and modified from [11]).

Alzheimer’s disease (AD), a neurodegenerative disease of devastating character, strikes over 40 million people worldwide [1]. It is the progressive and most common form of dementia characterized by cholinergic dysfunction, beta-amyloid plaques, and neurofibrillary tangles clustered in brain structures critical for memory and learning processes. A growing number of world elderly population makes this disease reaching epidemic proportions, causing serious human, social, and economic problems [2]. For that reason, AD is in the focus of pharmacological research. Two FDA approved drug classes for the symptomatic treatment of AD are acetylcholinesterase inhibitors (AChEIs) (donepezil, rivastigmine, and galantamine) and NMDA antagonist (memantine). Both classes of these drugs are administered individually or in combination and result in limited treatment success, partial or no response, high intersubject variability, often poor tolerability, and high costs. The side effects cover the broad palette of neurological and psychiatrical manifestations, gastrointestinal, dermatological and allergic disorders and electrolyte imbalance [3]. All these adverse and side effects raise the necessity for new symptomatic drugs that would have better efficacy-safety profile. One of the receptors that attracts the most attention for the potential individual or adjuvant therapy in AD is 5HT6 receptor [4,5,6,7]. 5HT6 receptor is serotonin postsynaptic G-protein metabotropic receptor coupled with the adenylyl cyclase second messenger system [8], which also activates ERK1/2 via Fyn-dependent pathway [2]. 5HT6 receptor is situated exclusively in CNS, on glutamatergic (hippocampus and throughout cerebral cortex), and GABAergic neurons (striatum, nc. Accumbens and olfactory bulb) [1,9,10]. Both neurotransmitter systems and neuroanatomical structures are constituents of memory and learning brain networks [9]. (Figure 1, adapted from [11]). The precise mechanisms of action of 5HT6 receptor antagonists are still unknown. The procognitive effect of 5HT6 receptor antagonists is probably due to the elevation of acetylcholine and glutamate concentrations in the brain by the deinhibition of GABA neurons [10,12]. Unexpected findings in which both 5HT6 receptor antagonists [13] and agonists [14] enhance the cognition in rodents suggest that 5HT6 receptors activate unknown biochemical pathways or act on distinct neuronal populations, inducing paradoxical procognitive effects [9].

On one side, the fact that (a) 5HT6 receptors regulate the balance between excitatory (glutamate) and inhibitory (GABA) signaling [15] and (b) they modulate synaptic plasticity and long-term potentiation in specific memory and learning regions [16] provides rational arguments for pharmacological targeting of the 5HT6 receptor. The brain tissue-specific distribution of this receptor is also the promising feature that 5HT6 pharmacomodulation will produce less peripheral adverse and side effects [1]. In a review by Khoury et al. [9], an overview was conducted of the current drug therapy for AD with recent clinical trials of the 5-HT6 receptor, with an emphasis on the importance of a better understanding of the mechanism of action of the 5-HT6 receptor and its ligands as partial or inverse agonists against AD [9].

Despite years of study efforts to develop therapies, there is no effective therapy available to cure AD or that considerably inhibits the progression of AD symptoms. Since 2003, although there are more than four hundred trials, no new drugs were approved. Most Phase II clinical trials do not succeed in Phase III often due to serious adverse effects or a lack of efficacy [17]. Eight agents listed in phase III in 2017, including the 5-HT6 inhibitors idalopirdine and intepirdine, failed in clinical trials as of 30 January 2018 [18].

Promising results in phase 2b study using intepirdine as adjunctive therapy to donepezil for AD dementia initiated its further examination in a phase 3 study: MINDSET [19]. However, it failed to achieve the assumed primary outcome as there were no statistically significant differences between intepirdine and placebo on the co-primary endpoints ADAS-Cog and ADCS-ADL. Therefore, the study was terminated as the main trial did not reach its primary outcome [20,21].

Only SUVN-502 achieved significant changes from a baseline in ADAS-Cog, especially in combination with donepezil and memantine [1,9,19,22].

SUVN-502 is a third 5-HT6 receptor antagonist that has been investigated for its safety and effectiveness in the phase IIa trial and in the background of donepezil and memantine [23] in patients with moderate AD. The SUVN-502 treatment, compared with placebo, improved behavioral symptoms as measured by neuropsychiatric inventory scores and reduced statistically significant the agitation/aggression scores. It was suggested that SUVN-502 might have a potential utility in the management of behavioral and psychological symptoms of dementia in AD [24]. In a press release of 16 August 2021, Suven Life sciences announced a Phase 3 Clinical Trial of NCE SUVN-502 (Masupirdine) to treat agitation and aggression in AD dementias. The enrollment of patients was expected from September 2021. This multi-center study is expected to be completed in about 36 months, with the result expected by the end of 2024 [25]. 

The lack of efficacy of many highly selective and specific drug candidates in treating many central nervous system diseases led to the development of multi-targeted drugs, among which include the multi-targeted drug candidate, AVN-101. AVN-101 is a very potent 5-HT7 receptor antagonist with slightly lower potency toward 5-HT6 [26].

AVN-101 is a potent 5-HT7 receptor antagonist that was well tolerated when taken orally at doses of up to 20 mg daily in the Phase I clinical study. AVN-101 was tested in clinical trials for Alzheimer’s disease [26]. 

Idalopirdine is a 5HT6 receptor antagonist (Saegis Pharmaceuticals) and was at first developed as a treatment for ‘Cognitive Impairment Associated with Schizophrenia’ (CIAS) [27]. After showing a positive effect on cognition, the drug was entered into clinical trials for use in Alzheimer’s dementia [1]. 

AVN-211 is a highly selective 5-HT6 receptor antagonist. Avineuro Pharmaceuticals has expressed intentions to start clinical trials of AVN-211 for Alzheimer’s disease in 2015 [28]. 

SGS-518 is also a potent and selective 5HT6 antagonist that is introduced by Piñeiro-Núñez et al. and Filla et al. [29,30].

The more recent clinical studies of 5HT6R antagonosts are mostly in ongoing or completed phase 1 clinical studies (HEC30654AcOH, ClinicalTrials.gov Identifier: NCT03655873, SB-742457, ClinicalTrials.gov Identifier: NCT00551772, PF-05212377, ClinicalTrials.gov Identifier: NCT01213355) [31,32,33].

Novel therapies prevent or delay the onset, slow the progression, or improve the symptoms associated with AD, and new targets are required for development of new drugs. The importance of finding new dementia treatments was also recognized by the G7 meeting, where it was stated that new efficient medications need to be developed by 2025 [34,35]. 

Natural products have been the primary source of medicines in all cultures. Success linked with screening progress and growing company collections was evident in providing the leads for many drug classes such as kinases and certain classes of the G-protein-coupled receptors (GPCRs) [36]. Natural products are the source of 25–50% of currently marketed drugs. The percentage of these drugs in anti-cancer and anti-infective diseases is even higher, reaching 66% [37]. Currently, two licensed anti-AD drugs (galantamine and rivastigmine) are based on plant-derived natural products, in addition to the fact that natural products targeting tau aggregation in AD pathogenesis are also reported [38].

Computer-aided or in silico designs have played a key role in the development of small molecule therapeutics for over three decades [39]. In silico techniques used in computational pharmacology allow better understanding and prediction of how drugs and compounds affect biological systems, enabling new methods to investigate the drug space and natural product’s “chemical space” [40,41]. The excellent and thorough review of Kelemen et al. of 5-HT6R as a target in AD with all the data regarding computational modeling tools in the field of 5-HT6R drug design, including ligand-based information, pharmacophore models, quantitative structure-activity relationship methods, crystal structure templates, homology models, along with the main goals and challenges in discovery new 5-HT6 antagonists, is very important and useful guide for the design of new 5-HT6R antagonists [42]. In a recent QSAR study, new potential arylsulfonamide-derived 5-HT6 receptor antagonists were proposed as new anti-AD ligands [43]. 

Virtual screening such as the in silico analog of high throughput screening of large compound databases is an integral part of the drug discovery process, which significantly reduces the time and cost of discovery of a new drug [44]. The availability of databases is essential for this strategy. One of the largest databases is Super Natural II, the first public, freely available web-based database of natural compounds that contains over 326,000 molecules [45]. The TCM Database@Taiwan is the world’s largest non-commercial database that contains compounds from traditional Chinese medicine with more than 20,000 pure compounds allowing free access to 3D compound structures [46]. The authors that have created and curated this database allowed the incorporation of its structures in ZINC. (https://zinc12.docking.org/pbcs/tcmnp, accessed on 31 March 2022). To date, the ZINC database [47,48] is the largest free 3D molecule database. The ZINC database contains over twenty million commercially available molecules. The database is freely available at http://zinc15.docking.org, accessed on 31 March 2022.

The aim of our study was to in silico investigate the ZINC database of natural products to find the potential candidates 5HT6 receptor antagonist for the treatment of AD dementia.

## 2. Results and Discussion

**Figure 2:**
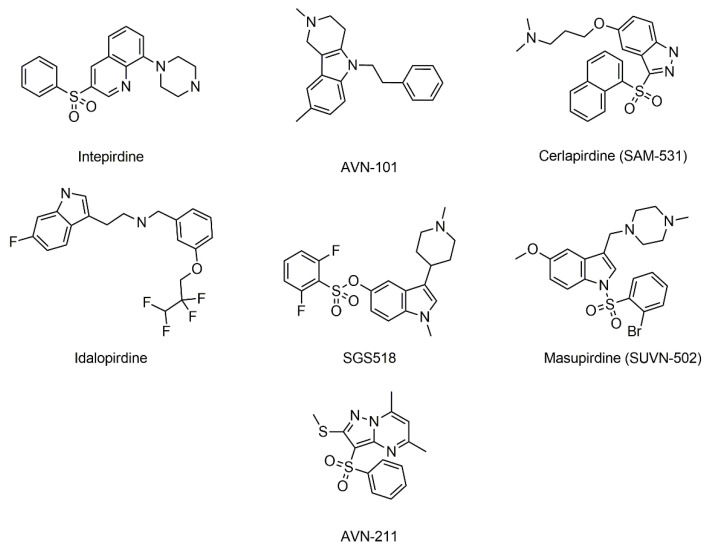
Structural formula of 5HT6 receptor ligand from literature.

First, we have applied EIIP/AQVN-based virtual screening of the ZINC Natural Product database. Since there is currently no approved drug antagonist for the 5HT6 receptor, the selected learning set consisted of 5HT6 receptor antagonists that are now in clinical testing as reported in the literature [42] and clinical candidates from the ChEMBL Target Report Card of Serotonin (5-HT) receptor antagonists [49]. The compounds from the learning set (Table 1, Figure 2) were inside the active domain with AQVN and EIIP values within the intervals of (2.51–2.90) and (0.009–0.09), respectively. The domain occupied by 5HT6 drug candidates was quite wide. In order to increase ligand specificity, we have chosen the two subdomains with drug candidates reported to reach the furthest phase of a clinical trial [42] with EIIP/AQVN values 2.55/0.09 and 2.99/0.009 and the third domain with EIIP/AQVN 2.72/0.055 that occupies serotonin, the natural ligand of 5HT6 receptor. The specified domains with AQVN and EIIP values were selected as a criterion for the selection of compounds representing potential candidate 5HT6 antagonists. By applying the EIIP/AQVN-based virtual screening criterion, 789 candidates were chosen from the ZINC Natural Product database (http://zinc15.docking.org/, accessed on 31 March 2022) [50].

After PCA virtual screening, we selected ten of the most similar compounds to the learning set from each EIIP/AQVN range, which was further subjected to molecular docking. The data on PCA model are available in Supplementary Materials. 

**Figure 3:**
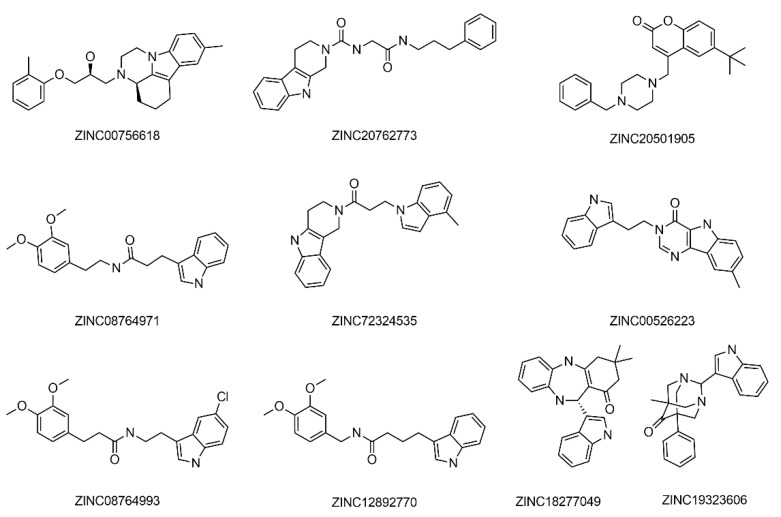
Structural formula of selected 5HT6 receptor candidates from ZINC database.

Furthermore, after the docking process, we selected ten best candidates (Table 2, Figure 3) based on their docking score, i.e., the best balance between calculated free binding energy and favorable and non-favorable protein-ligand interactions. 

**Figure 4:**
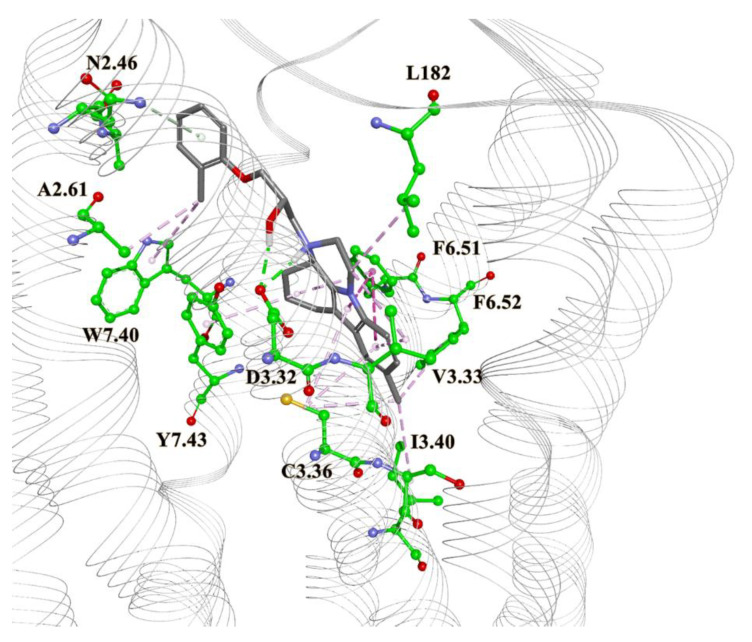
Homology model of 5HT6 receptor with docked best candidate compound ZINC00756618 and interacting AA residues. Green lines: hydrogen bonds; purple: aromatic interactions; grey: hydrophobic interactions; orange: anion—PI interaction.

**Figure 5:**
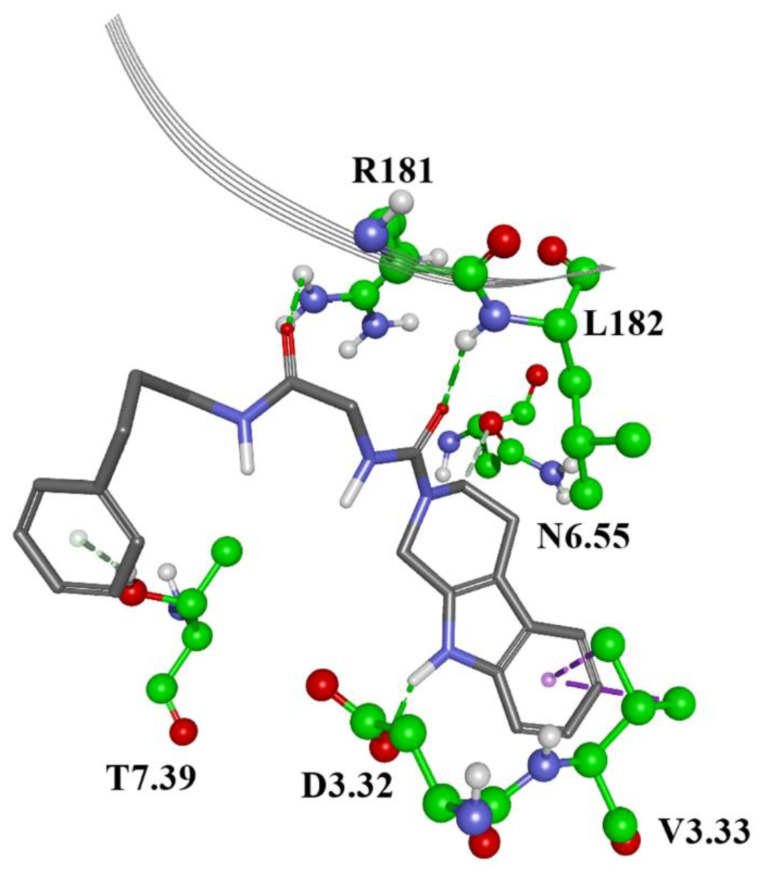
Homology model of 5HT6 receptor with docked second best candidate compound ZINC20762773 and interacting AA residues. Green lines: hydrogen bonds; purple: aromatic interactions; grey: hydrophobic interactions; orange: salt bridge or anion—PI interaction.

The results of the docking process showed that molecules form protein–ligand interactions with the following residues in 5HT6 receptor: Asp 106 (D3.32), as an anchor AA residue, by either salt bridge or hydrogen bonding. Ser 193 (S5.43), Thr 196 (T5.46), Asn 288 (N6.55), and Tyr Y7.42 provide additional hydrogen bonding with the receptor. Residues Trp 102 (W3.28), Val 107 (V3.33), Cys 110(C3.36), Trp 281(W6.48), Phe 284 (F6.51), Phe 285 (F6.52), Asp 303 (D7.36), Trp 307 (W7.40), and L157 from ECL2 form hydrophobic or aromatic interactions with a ligand molecule. Those residues were earlier reported as necessary for antagonistic behaviors [51,52,53,54,55,56,57]. Moreover, other reported interaction residues include Asn 86 (N2.64) [58], Ser185 (S5.36), Thr103 (T3.29) [59], Asp 303 (D7.36), Phe 188 (F5.38), and Ala 157 (A4.56) [60]. As a control, we docked the literature antagonists (Figure 1, Table 1). The 2D binding patterns obtained by GOLD are presented on Figures S1–S6 in Supplementary Materials. Figure 4 and Figure 5 present docked conformations of the compounds ZINC00756618 and ZINC20762773, respectively.

**Figure 6:**
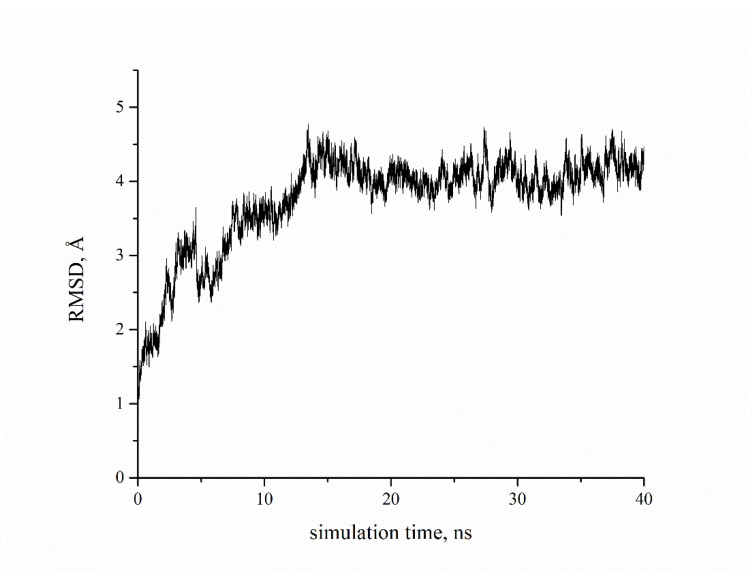
RMSD of 5HT6 backbone atoms during the production phase of complex with ZINC00756618.

**Figure 7:**
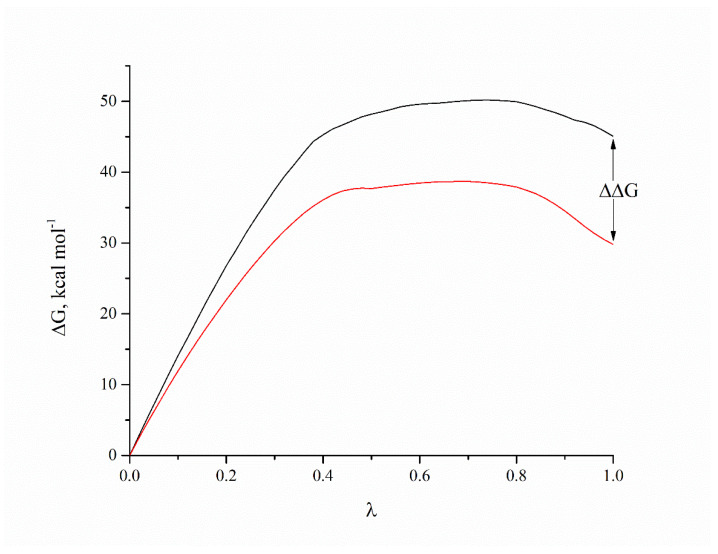
FEP results for bound (red line) and unbound (black line) conformation of the 5HT6–ZINC00756618 complex.

The first and second compounds are the best candidates, and they belong to the AQVN descriptor range 2.55. The third compound is the best among those belonging to the AQVN descriptor range 2.77. As mentioned, the anchor AA residue D3.32 forms either hydrogen bonds or salt bridges with protonated or charged nitrogen atoms, while other residues form appropriate interactions depending on the ligand structure. By comparing the GOLD scores (dimensionless quantity) of our candidates to the known ligands from literature (Table 2), they fall in the almost same range, (32.08–47.13) for the candidates and (32.3–42.36) for the literature compounds. Autodock Vina results confirmed the conformations obtained from GOLD (Figures S7 and S8, Supplementary Material) and binding energies are in good correlation except for a few discrepancies. Molecular dynamics simulation of the 5HT6 best candidate complex showed the stability of the receptor, reaching RMSD values around 4 Å after 12ns (Figure 6). In addition, the stabilities of ligand’s conformation in the binding site (Figure S9) and salt bridge between protonated nitrogen in ligand and D3.32 of the receptor (Figure S10) are presented. For the calculation of binding free energy, we chose the FEP thermodynamic integration approach due to the impossibility to perform metadynamics simulation, as the extracellular loop of the receptor hindered ligand’s pulling out of the binding site. After double annihilation consisting of forward-backward FEP for both bound and unbound ligand states, using SOS energy averaging in VMD, we calculated the binding free energy of the best candidate compound (Figure 7).

ΔΔG = ΔG_unbound_ − ΔG_bound_ = 45.06–29.79 = 15.27 kcal/mol, i.e., −15.27 kcal/mol.

Taking into account the size of the system and error, this is an acceptable correlation with binding free energy obtained from Autodock Vina (−13.13 kcal/mol), which indicates the nanomolar activity of our compound (calculated from relation ΔG = RTlnK, where R = 8.314 J mol^−1^ K^−1^, T = 298 K).

The drug-likeness of a compound was assessed according to Lipinski’s Rule of Five [61], which considers molecular weight (<500 Da), the number of hydrogen-bond acceptors (≤10) and donors (≤5), and octanol/water partition coefficient (≤5), and Jorgensen’s rule of three [62], which regards logS (>−5.7), PCaco (>22 nm/s), and primary metabolites (PM) (<7). The violations of these rules are essential for the optimization of biologically active compounds and should not be more than 1.

Table 3 presents predicted the ADMET properties of candidate compounds with the following parameters: molecular weight (MW), number of rotatable bonds (RB), dipole moment (DM), molecular volume (MV), number of hydrogen donors (DHB), number of hydrogen acceptors (AHB), polar surface area (PSA), octanol/water partition coefficient (log P), aqueous solubility (log S), apparent Caco-2 cell permeability (PCaco), number of likely primer metabolic reactions (PM), percentage of human oral absorption (%HOA), violations of rules of three (VRT) and five (VRF), blood–brain barrier permeability parameter (QPlogBB) [62], and Multi-Parameter Optimisation score for drugs targeted at the Central Nervous System (CNS MPO) [63]. ADME parameters are presented along with the violations of Lipinski’s and Jorgensen’s rules. All compounds follow both rules, causing no more than one violation. Thus, according to the ADMET properties, all compounds may possess a good pharmacokinetic profile and may be a good candidate for the treatment of AD dementia. Table 4 presents QPlogBB and CNS MPO values for the literature antagonists for comparison.

Since 1993, the year of the discovery of the 5HT6 receptor and the following period of experiments on 5HT6 receptor antagonists, the 5HT6 receptor deserves special attention in the treatment of cognitive deficits [2,10]. In addition to its procognitive effect, the importance of 5HT6 targeting in AD is emphasized by the fact that this receptor is also involved in affective disorders, anxiety, depression, epilepsy, and obesity, pathologies that often accompany and complicate the clinical status of Alzheimer patients. Even though a substantial number of compounds with antagonist effect on 5HT6 receptors was successful in phase I and II of clinical trials (idalopirdine, intepirdine, latrepirdine, and SAM-531), they did not succeed in Phase III, for various reasons, often owing to serious adverse effects or lack of therapeutic efficacy [17]. As a reason why no 5-HT6R ligand has progressed through all clinical stages and the reason for failure in clinical trials is that candidates are unable to cross the blood–brain barrier [43]. An example of the successful drug on the way to be FDA approved is SUVN-502 [9,12,22]. The triple combination of SUVN-502 with donepezil and memantine synergistically increased the acetylcholine levels in the ventral hippocampus [12]. It is important to stress that SUVN-502 is the closest to EIIP/AQVN values to serotonin, the natural ligand of the 5HT6 receptor. Recently, new benzimidazole-based compounds have been developed as a new class of 5-HT6R antagonists with favorable in vivo properties. Supporting the fact that 5HT6 receptor antagonists represent a step toward the identification of new cognitive enhancers for the treatment of AD, the development of new 5-HT6R antagonists is still required to validate these molecules as a drug class against AD [61].

For in silico approaches or computer-aided drug, the design has provided a large number of opportunities for the identification of novel potential lead compounds against many diseases, including AD, saving the cost and time of performing clinical trials and increasing the success rate of new drugs [64]. These approaches comprise the conformational modeling of small molecules, macromolecules and their docked complexes, calculation descriptors, and the independent molecular properties, followed by model constructions, the design of novel molecules, and the prediction of the potential of bioactive compounds [65]. 

Natural products are promising potential sources for novel compounds that can meet the criteria of better efficacy and better tolerability in the treatment of AD. In silico approaches could be of significant help as there remains a significant need for new 5-HT6R leads and drug candidates with the potential to improve cognitive deficits in AD.

It was shown earlier for molecular targets in various pathologies that small molecules with similar AQVN and EIIP values interact with the common therapeutic target [66]. This has led to the definition of the EIIP/AQVN criteria for the virtual screening of molecular libraries for compounds with similar treatment properties [67]. Prior studies have verified the EIIP/AQVN approach to be useful in identifying inhibitors against various viral and chronic disease targets [68,69] that were also confirmed experimentally [70,71]. 

The EIIP/AQVN approach in the previous drug database screening revealed potential candidates for the pharmacotherapy of neurocardiovascular diseases and chronic tinnitus [72,73]. In this study, an in silico EIIP/AQVN-based filter was applied as the first for the selection of 5HT6 receptor antagonists during the virtual screening process. As a result, 789 candidates were chosen from the ZINC—natural product database. This step was followed by PCA analysis and, finally, molecular docking. The screening identified ten best candidates as the most promising 5HT6 receptor antagonist. Our selected candidates show good CNS drug ability properties. There is no known activity for the ten virtual hits in the ZINC database or other databases linked to ZINC.

NPBS (Natural Products & Biological Sources) database is a chemical database connecting natural products and biological sources that is manually curated from the literature [74]. The biological sources cover diverse plant, bacterial, fungal, and marine species. In the NPBS database, we did not find a biological source for any of the ten compounds. Furthermore, we investigated the natural origin of the ten virtual hits in the KNApSAcK Family Database [75], which contains data on compounds linked to information on the plant family and species from which the compound was isolated. However, we did not find any data about plant families or species for the ten proposed hit compounds. The proposed candidates will be evaluated in further experimental testing.

## 3. Materials and Methods

A library of natural products that contain 1 × 10^5^ compounds was downloaded from the ZINC database in the processed format [48]. The EIIP/AQVN approach was employed for in silico screening of the ZINC—natural product database and then proceeded by PCA analysis and molecular docking for the identification of potential 5HT6 receptor antagonist. 

### 3.1. EIIP/AQVN 

The EIIP for organic molecules can be determined by the following simple equation derived from the “general model pseudopotential [67]:

EIIP = 0.25 × Z* × sin (1.04 × π × Z*)/2π,(1)

where Z* is the average quasi valence number (AQVN) determined by the following:

Z* = ∑m (ni × Zi/N),(2)

where Zi is the valence number of the ith atomic component, ni is the number of atoms of the ith component, m is the number of atomic components in the molecule, and N is the total number of atoms. EIIP values calculated according to Equations (1) and (2) are expressed in Rydberg units (Ry).

### 3.2. Zinc Ligand Database and Preparation

The structures of all candidate ligands were taken from the ZINC database [48] in sdf format, which is already geometrically optimized and protonated at physiological pH. The total number of ligands from all three EIIP/AQVN ranges was 789. 

Ligands for the learning sets were downloaded from the CHEMBL database [76], including receptor ID CHEMBL3371 containing 3292 active compounds and containing Ki values in the range from 0.04 nM to 30 μM. The set was randomly sampled, resulting in 100 compound learning set for virtual screening. Molecules were converted from SMILES to sdf format.

### 3.3. PCA Analysis of Virtual Screening

The obtained learning set from the previous step was imported, and molecules were aligned towards the principal moment of inertia axis and protonated on physiological pH (7.4). The candidate molecules were also imported for the next virtual screening step. MIF descriptors were computed for all molecules. The computation method for descriptor generation was GRID with step 0.5. Applied probes (mapped regions of molecule surface) were DRY (hydrophobic interactions), O (hydrogen bond acceptor), N1 (hydrogen bond donor), and TIP (molecular shape descriptor). The discretization method was AMANDA with a scale factor of 0.55. The encoding method was MACC2, and the weights were the following: DRY: −0.5; O: −2.6; N1: −4.2; and TIP: −0.75. Based on the learning set, the PCA model was built, consisting of five principal components. The explained variance was 64.84%. The virtual screening of compound candidates was carried by the use of the shortest centroid distance. The calculation was carried in Pentacle software version 1.06 for Linux [77,78,79]. From each of the three candidate groups, ten of the most similar molecules were selected and further submitted to molecular docking.

### 3.4. Receptor Preparation

The homology model of the human 5HT6 receptor was downloaded from the GPCRdb database [80] in its intermediate form. The receptor was protonated at physiological pH 7.4. All receptor amino acid residues (AA) were numbered in Ballesteros-Weinstein nomenclature [81]. 

### 3.5. Molecular Docking

Chemscore kinase protocol was used for the molecular docking process [82,83]. The binding site for molecular docking was chosen as a 15 Å radius sphere, with the center in the carbon atom of the D3.32 carboxyl group. The (x,y,z) center of the grid box was (−25.9, 5.82, 18.04). As a scoring function was used, the ChemScore. Search Efficiency was set to 200%. The best ten candidates, based on score and specific receptor–ligand interaction criteria, were selected. The calculations were carried in GOLD software [84]. Molecular docking was also carried in newly developed Autodock Vina 1.2.0 [85] using Autodock4 forcefield. Receptor maps were prepared using Autogrid 4. Exhaustiveness was set to 250. Both receptor and ligands were prepared in ADT Tools 1.5.6 [86] 

For intermolecular interaction identification, the hydrogen bonds were recognized if a maximum distance between the donor (D) and a hydrogen atom (H), D · · · H, was 3.4 Å and had an angle D-H-A between 90° and 180°. For a salt bridge, a maximum distance between the charged atoms was 4 Å. For π–alkyl interactions, the maximum distance between the centroid of the aromatic ring and the C-atom of the alkyl group was 4 Å and angle 45°. For π–π interactions, the maximum distance between centroids was 6 Å, and the values of theta and gamma angles were 50° and 35° for stacked and 30° and 55° for T-shaped conformation, respectively. For π–cation interactions, the maximum distance between cation and centroid was 4 Å and angle 40°.

### 3.6. Molecular Dynamics Simulations and Binding Free Energy Calculations

The 5HT6-ligand complex was inserted into a 70 × 70 Å 2-oleoyl-1-palmitoyl-sn-glyecro-3-phosphocholine (POPC) lipid bilayer, created in VMD 1.9.3 [87]. A 20 Å water layer was added from the both sides of the z-axis. Bad-contact water molecules were removed from the lipid membrane bilayer using the appropriate tcl script. Additionally, the system was neutralized with 0.15 M NaCl, resulting in a 61,203 atoms ensemble. The system was subjected to a 10,000-step energy minimization, 250 NVE ps equilibration, and 40 ns NPT MD production. Pressure and temperature were set to 1 bar and 310 K, respectively, using a Berendsen thermostat, and the applied integration step was 1 fs. In all simulations, periodic boundary conditions with particle-mesh Ewald calculations were implemented. The cut-off was set to 12 Å. A CHARMM36 force field was used [88]. 

Binding free energy was calculated using Free Energy Perturbation (FEP) approach. Thermodynamic integration was carried for bound and unbound ligand conformation (double annihilation), both forward and backward, using soft-core potential approach. Alchemical Van der Waals shift coefficient was set to 6.0. Each λ step of simulation consisted of 100 ps alchemical equilibration, followed by 400 ps of production. The simulation was set from λ 0 to 1, with step 0.02, in 50 cycles. The simulation took 137,439 s for bound and 27,940 s for unbound form @ Intel i7-10700KF @3.80 GHz and NVIDIA GeForce 1080 Ti.

All simulations were carried in NAMD 3.0 alpha, which is able to perform CUDA accelerated FEP calculations [89]. Results were analyzed in VMD 1.9.3 Analyse FEP simulation extension with an SOS (simple-overlap sampling) estimator.

### 3.7. ADME/Toxicity Prediction

ADMET parameters of the natural compounds (1–10) were calculated with the help of QikProp software running in normal mode [90,91]. CNS MPO values were calculated using MarvinSketch 22.9 [92]. 

## 4. Conclusions

Natural compounds present an important treatment source of novel, potent, and selective candidates for treating the cognitive deficit due to AD. The ZINC Natural Product database was virtually screened for candidate antagonists of 5HT6 receptors. The ten most promising candidate compounds were selected as potential candidates. The results of our study will be confirmed experimentally in further research studies that will show whether these compounds represent suitable preliminary candidates to alleviate the heavy burden of Alzheimer’s disease to patients, families, and the public health system. 

## Figures and Tables

**Table 1:** List of literature compounds for 5HT6 receptor, with their docking scores.

No	Name	Gold Score	Autodock Vina Binding Energy (kcal/mol)	AQVN	EIIP
1	Intepirdine	42.36	−5.94	2.909	0.009
2	Cerlapirdine (SAM-531)	36.63	−8.13	2.88	0.000
3	Idalopirdine	34.59	−9.193	2.55	0.089
4	SGS518	33.92	−9.366	2.78	0.035
5	Masupirdine (SUVN-502)	35.55	−10.52	2.79	0.032
6	AVN-211	32.30	−5.335	3.08	0.073

**Table 2:** List of the best candidate compounds for 5HT6 receptor, based on their docking scores.

No	Name	Gold Score	Autodock Vina Binding Energy (kcal/mol)	AQVN	EIIP
1	ZINC00756618	43.17	−13.13	2.55	0.090
2	ZINC20762773	39.05	−11.57	2.55	0.090
3	ZINC20501905	35.01	−11.47	2.55	0.090
4	ZINC08764971	34.65	−9.11	2.72	0.055
5	ZINC72324535	34.48	−10.7	2.72	0.055
6	ZINC00526223	34.38	−9.135	2.99	0.009
7	ZINC08764993	33.48	−9.371	2.72	0.055
8	ZINC12892770	32.94	−8.995	2.72	0.055
9	ZINC18277049	32.65	−9.072	2.72	0.055
10	ZINC19323606	32.08	−10.04	2.72	0.055

**Table 3:** Calculated absorption, distribution, metabolism, elimination, and toxicity (ADMET) parameters of the compounds.

Compound	MW	RB	DM	MV	DHB	AHB	PSA	logP	logS	PCaco	PM	%HOA	VRF	VRT	QPlogBB	CNS MPO
ZINC00756618	390.5	6	3.2	1304.9	1	4.45	31.5	5.24	−5.68	1416	8	100	1	1	0.33	3.22
ZINC20762773	390.5	6	8.1	1287.3	2.25	3.75	86.8	3.98	−4.91	348	4	95	0	0	−0.92	4.81
ZINC20501905	390.5	5	6.7	1317.7	0	6.5	46.9	3.48	−3.34	125	4	85	0	0	0.29	3.22
ZINC08764971	352.4	8	5.9	1133.5	2	4	65.4	3.74	−3.92	1475	5	100	0	0	−0.55	4.73
ZINC72324535	357.5	3	6.3	1182.2	1	3	45.3	4.82	−5.97	1440	4	100	0	1	−0.28	4.53
ZINC00526223	342.4	3	2.6	1102.3	2	4	69.6	3.99	−5.73	839	3	100	0	1	−0.75	4.41
ZINC08764993	386.8	8	8.7	1202.5	2	4	63.3	4.46	−5.39	1724	5	100	0	0	−0.39	3.94
ZINC12892770	352.4	8	5.1	1179.7	2	4	68.2	4.03	−4.73	1159	5	100	0	0	−0.69	4.58
ZINC18277049	357.4	0	6.8	1087.1	2	2.5	62.2	4.61	−5.69	1541	8	100	0	1	−0.16	4.08
ZINC19323606	357.4	0	4.7	1082.3	1	6	43.7	2.55	−2.48	175	2	82	0	0	0.84	4.14

**MW**: Molecular weight; **RB**: Number of rotatable bonds; **DM**: computed dipole moment; **MV**: total solvent-accessible volume; **DHB**: estimated number of hydrogen-bond donors; **AHB**: estimated number of hydrogen-bond acceptors; **PSA**: van der Waals surface area of polar nitrogen and oxygen atoms and carbonyl carbon atoms; **logP**: predicted octanol/water partition coefficient; **log S**: predicted aqueous solubility; **PCaco**: predicted apparent Caco-2 cell permeability; **PM**: number of likely metabolic reactions; **% HOA**: predicted human oral absorption percentage; **VRF**: number of violations of Lipinski rule of five (the rules are as follows: MW< 500, log P < 5, DHB ≤ 5, AHB ≤ 10, positive PSA value); **VRT**: number of violations of Jorgensen rule of three (the rules are as follows: log S > −5.7, PCaco > 22 nm/s, PM < 7));QP log BB, brain/blood (good values to range 95% of drugs are from −3 to 1.2); CNS MPO. The result is a decimal number between 0 and 6, with “ideal” molecules scoring 6.

**Table 4:** List of literature compounds for 5HT6 receptor, with their calculated QPlogBB and CNS MPO values.

No	Name	QPlogBB	CNS MPO
1	Intepirdine	−0.25	5.36
2	Cerlapirdine (SAM-531)	−0.74	4.89
3	Idalopirdine	0.53	3.07
4	SGS518	0.13	3.41
5	Masupirdine (SUVN-502)	0.46	4.52
6	AVN-211	−0.29	5.34

## Data Availability

Not applicable.

## Supplementary Materials

The following supporting information can be downloaded at: https://www.mdpi.com/article/10.3390/molecules27092626/s1, Figure S1. Binding pattern of Intepirdine in the active site of 5HT6 receptor; Figure S2. Binding pattern of Cerlapirdine in the active site of 5HT6 receptor; Figure S3. Binding pattern of Idalopirdine in the active site of 5HT6 receptor; Figure S4. Binding pattern of SG518 in the active site of 5HT6 receptor; Figure S5. Binding pattern of Masupirdine in the active site of 5HT6 receptor; Figure S6. Binding pattern of AVN-211 in the active site of 5HT6 receptor; Figure S7. Binding modes of compound ZINC00756618, obtained by GOLD (green) and Autodock Vina (cyan); Figure S8. Binding modes of compound ZINC2076277, obtained by GOLD (green) and Autodock Vina (cyan); Figure S9. RMSD of ZINC00756618 in the binding site of 5HT6 receptor during 40ns production phase of MD simulation; Figure S10. Salt bridge in 5HT6—ZINC00756618 complex during 40ns production phase simulation, presented as interactions of NH+ (ligand) and oxygens from COO- group from D3.32. Table S1: List of compounds from AQVN group values 2.55 with centroid distance from the learning set; Table S2: List of compounds from AQVN group values 2.72 with centroid distance from the learning set; Table S3: List of compounds from AQVN group values 2.99 with centroid distance the from learning set; Table S4: 5HT6 compounds learning set from ChEMBL database.

## Sample Availability

Samples of the compounds are not available from the authors.
