# Supplementary material for: In Silico Screening of Natural Compounds for Candidates 5HT6 Receptor Antagonists against Alzheimer’s Disease
Source: Molecules. 2022 Apr 19;27(9):2626. doi: 10.3390/molecules27092626 (PMC9101541; doi:10.3390/molecules27092626)
Supplement: Supplementary file 1 [file molecules-27-02626-s001.zip › molecules-1599972-supplementary.pdf]

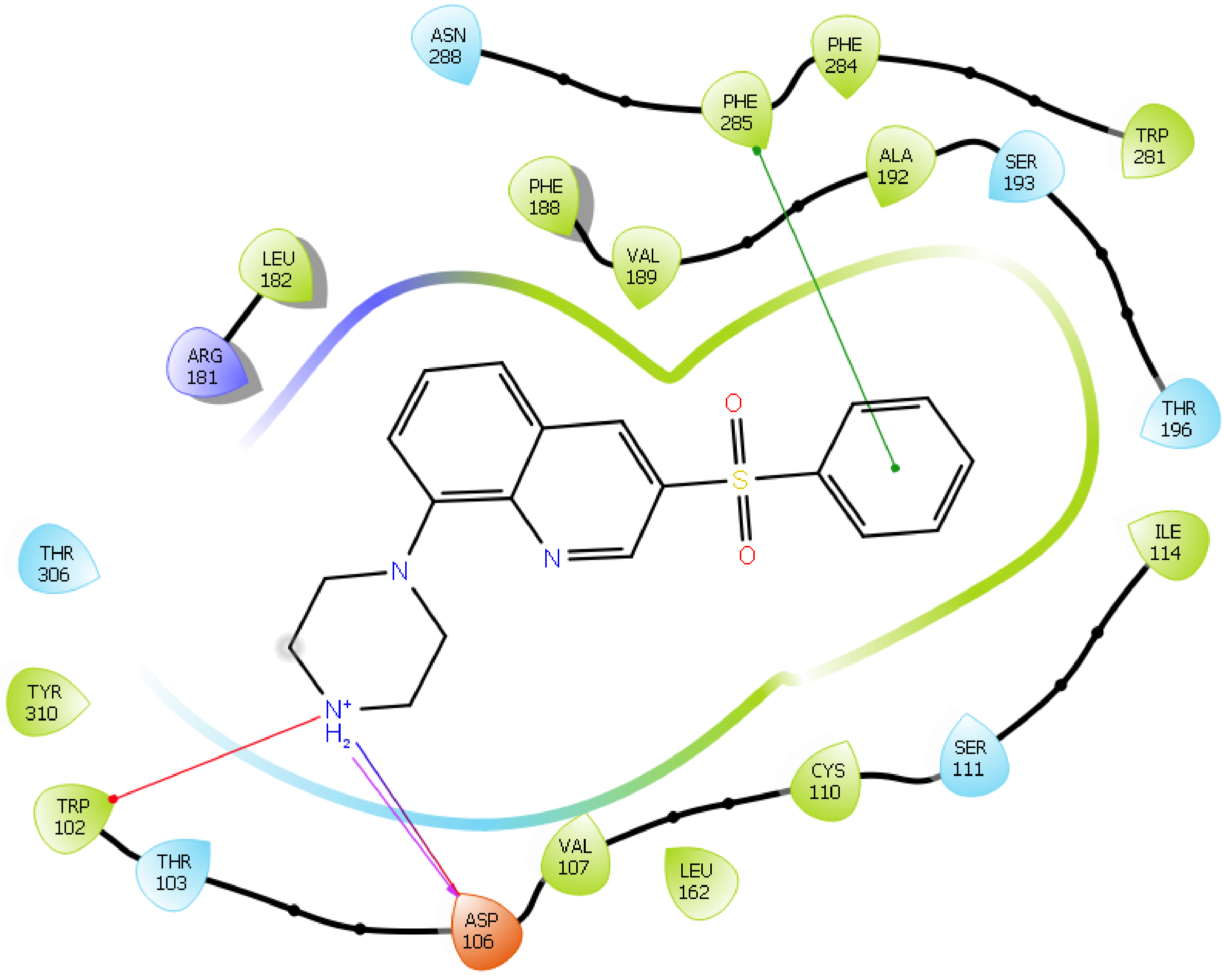

Figure S1. Binding pattern of Intepirdine in the active site of 5HT6 receptor

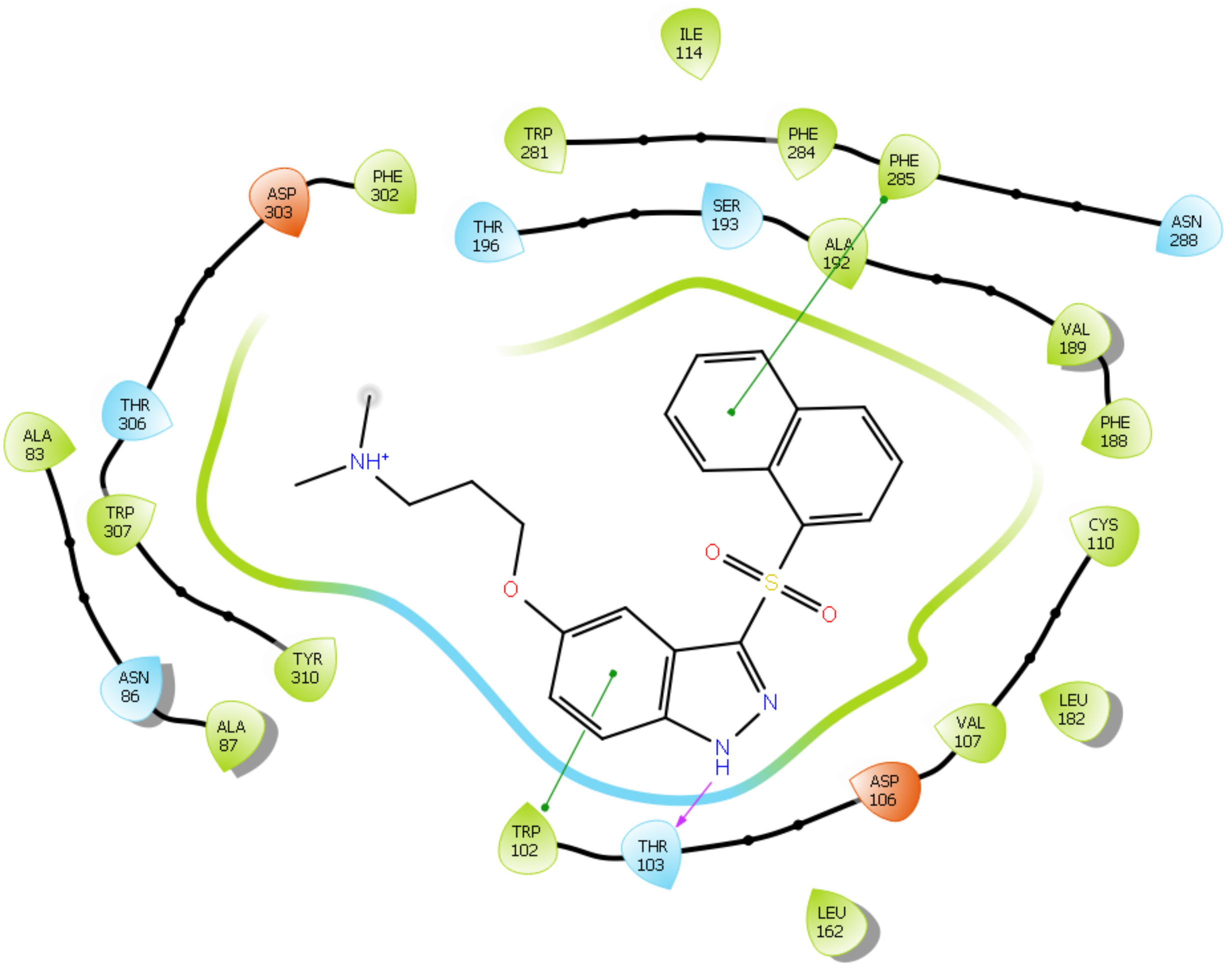

Figure S2. Binding pattern of Cerlapirdine in the active site of 5HT6 receptor

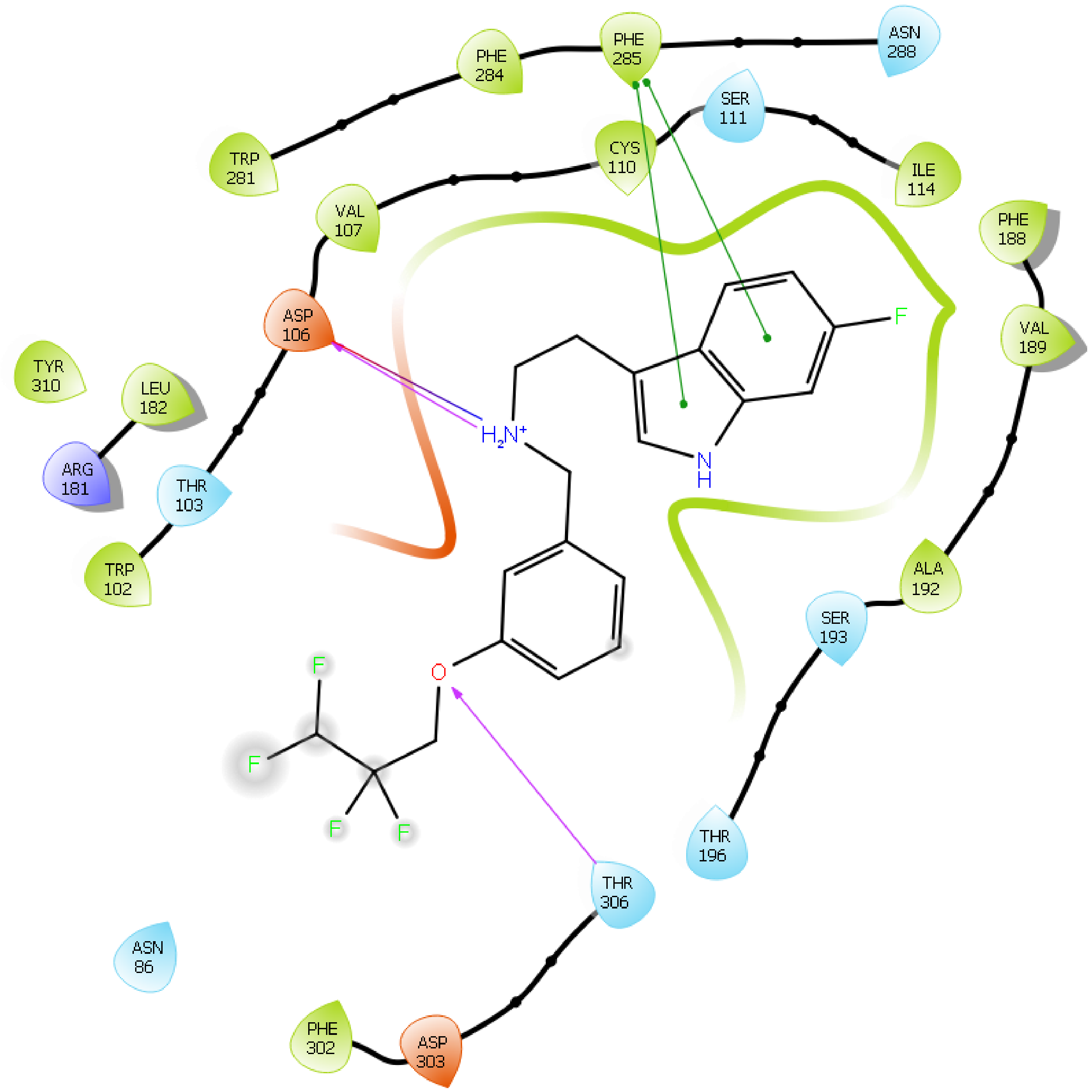

Figure S3. Binding pattern of Idalopirdine in the active site of 5HT6 receptor

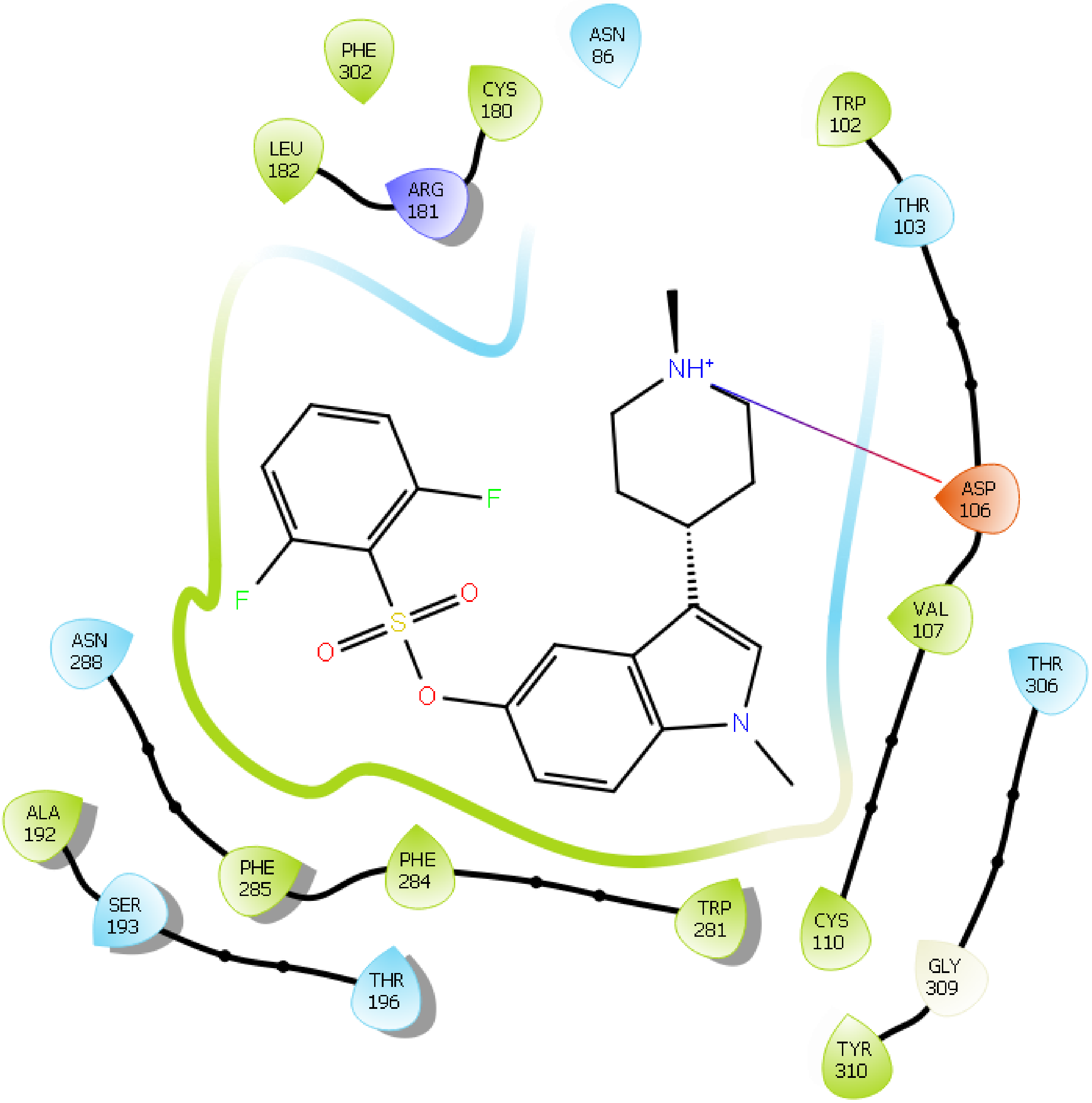

Figure S4. Binding pattern of SG518 in the active site of 5HT6 receptor

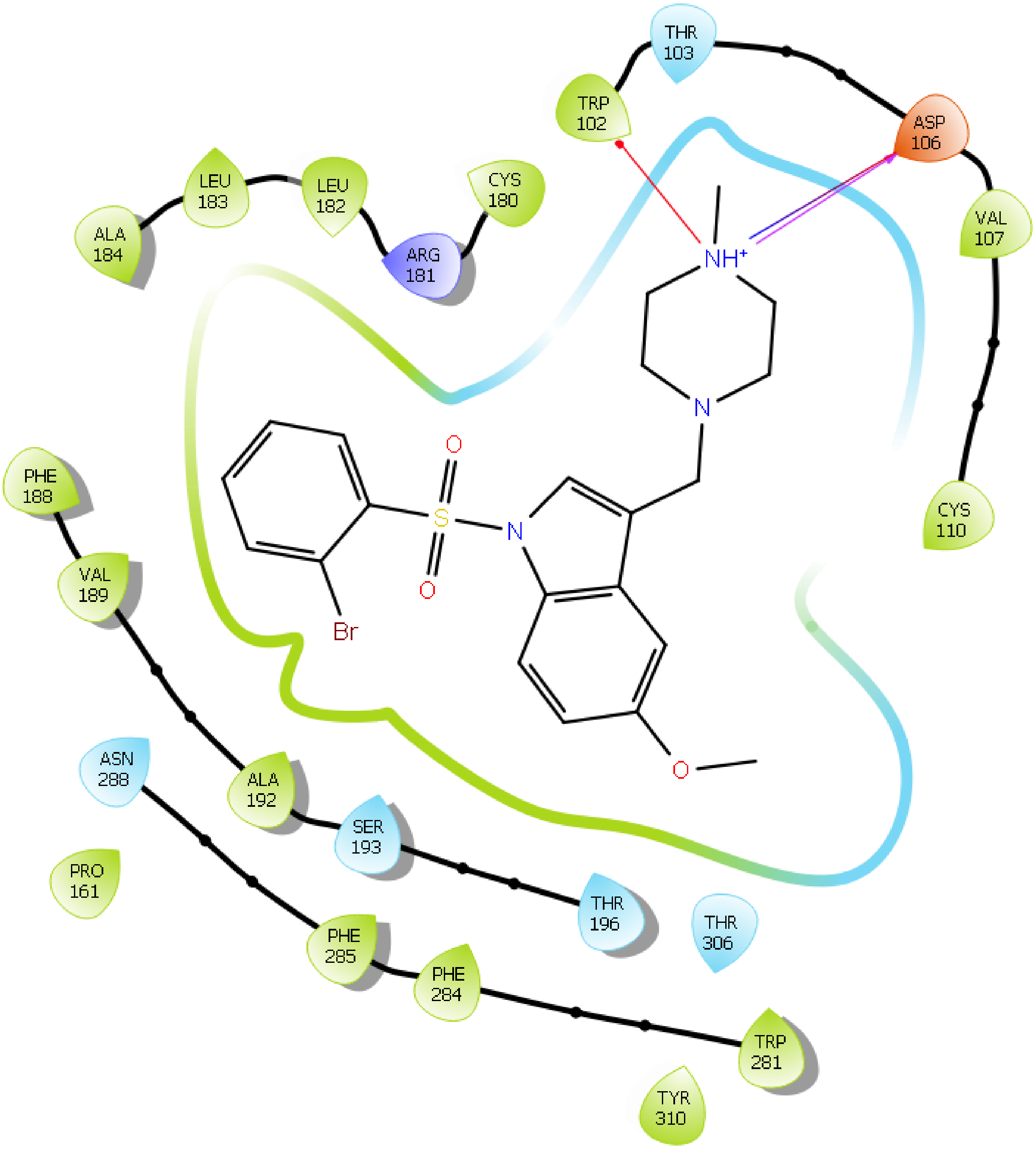

Figure S5. Binding pattern of Masupirdine in the active site of 5HT6 receptor

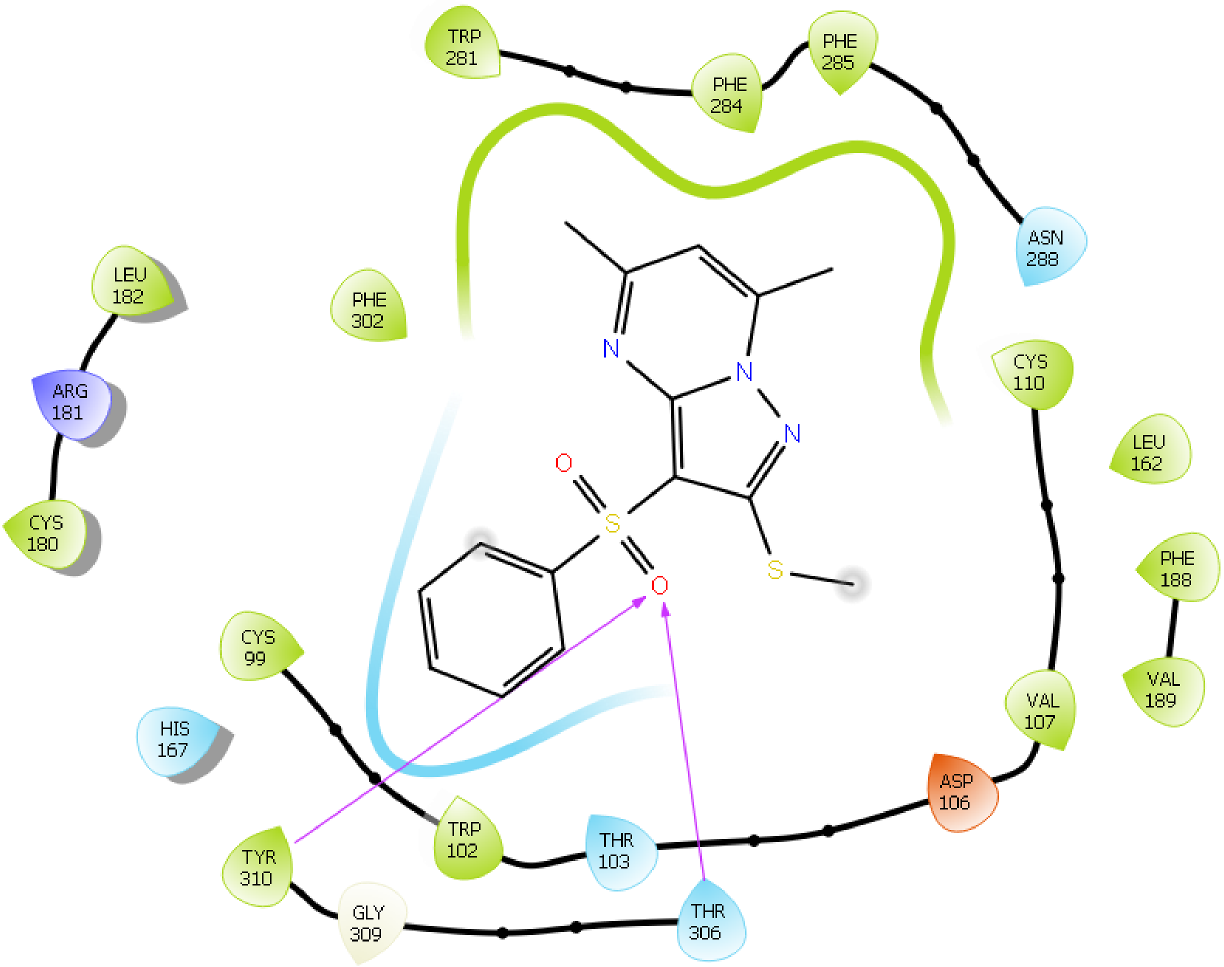

Figure S6. Binding pattern of AVN-211 in the active site of 5HT6 receptor

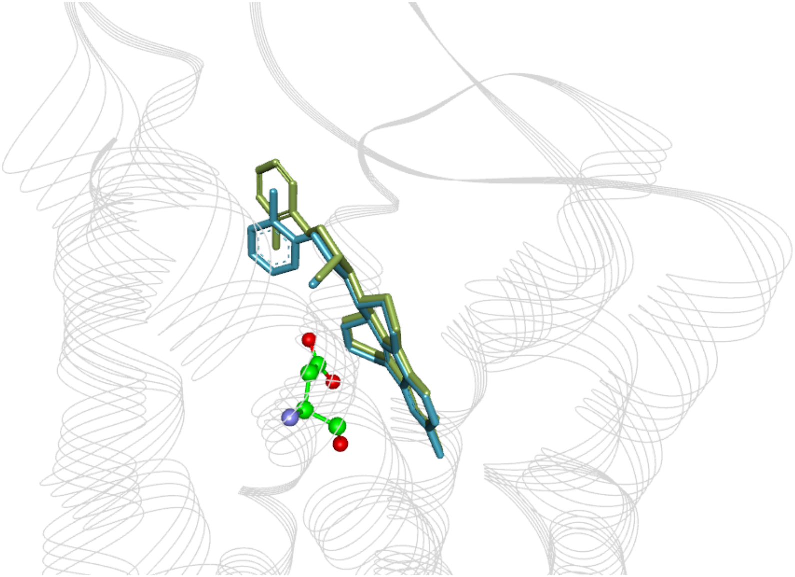

Figure S7. Binding modes of compound ZINC00756618, obtained by GOLD (green) and Autodock Vina (cyan)

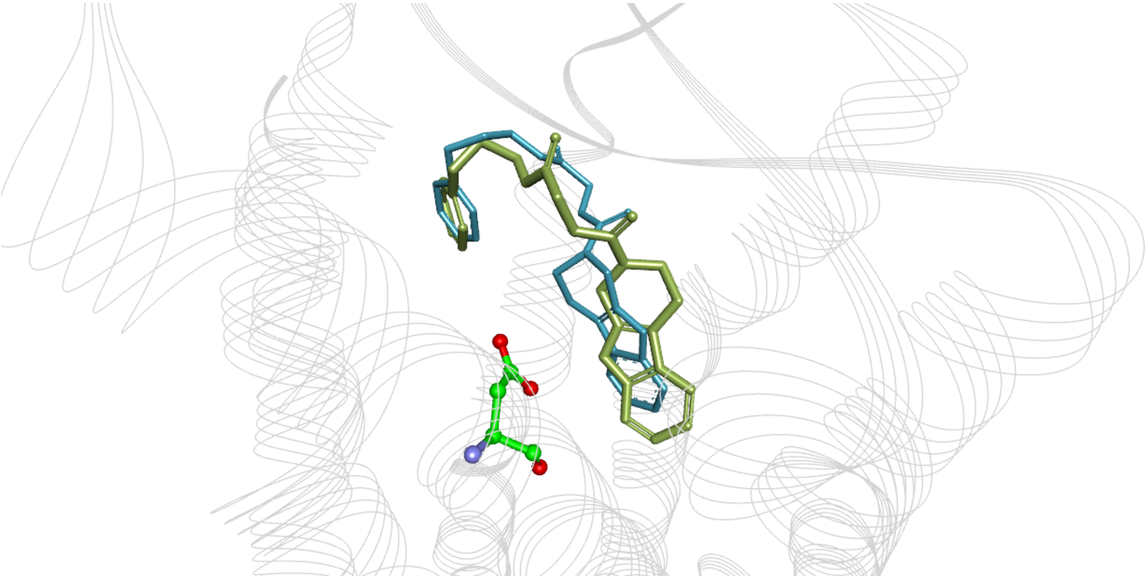

Figure S8. Binding modes of compound ZINC2076277, obtained by GOLD (green) and Autodock Vina (cyan)

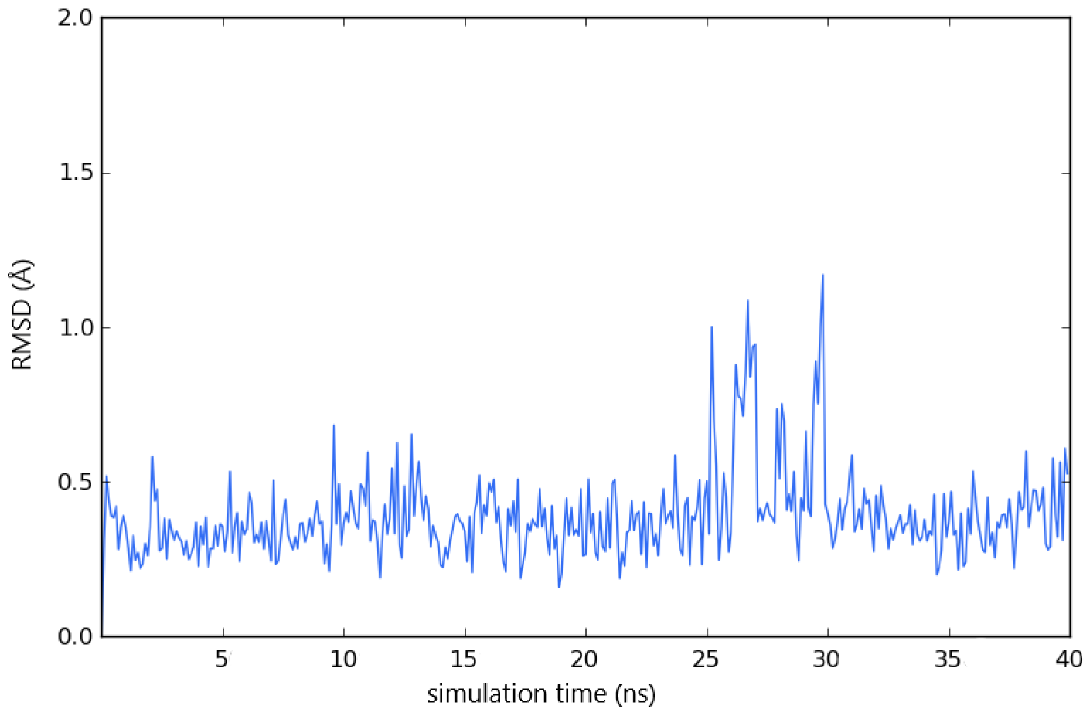

Figure S9. RMSD of ZINC00756618 in the binding site of 5HT6 receptor during 40ns production phase of MD simulation

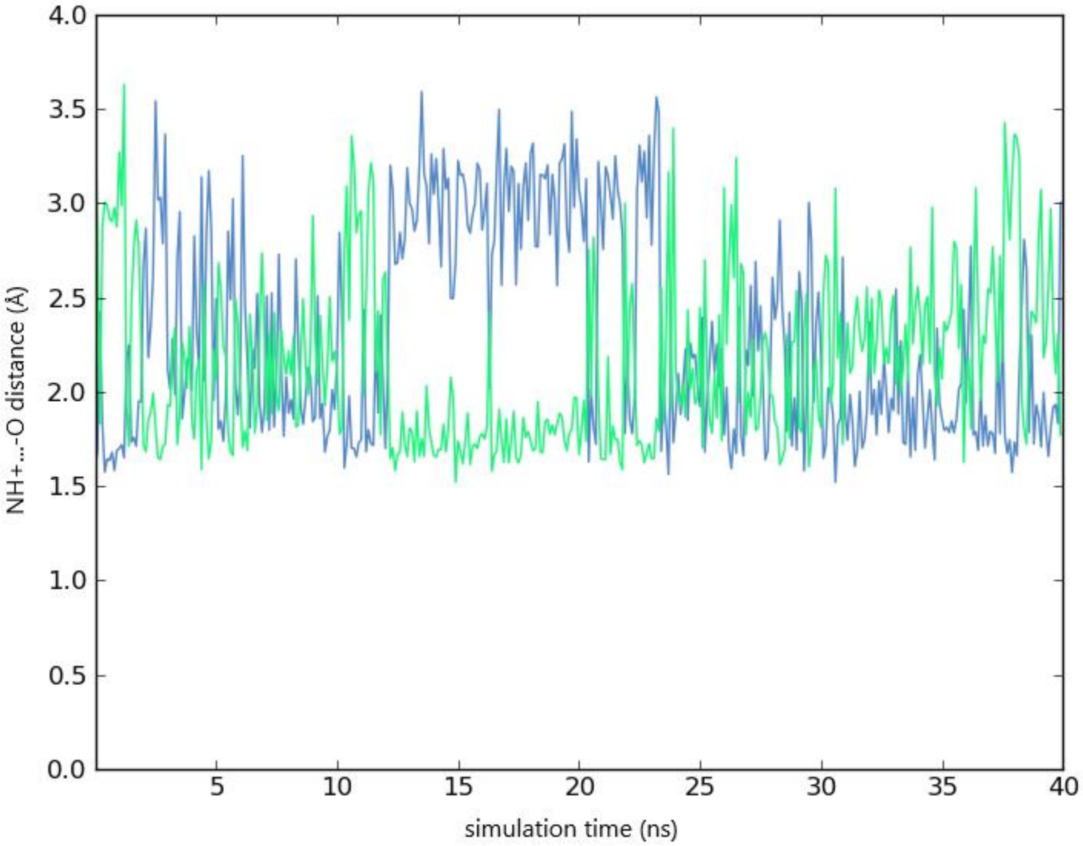

Figure S10. Salt bridge in 5HT6 - ZINC00756618 complex during 40ns production phase simulation, presented as interactions of NH+ (ligand) and oxygens from COO^{-} group from D3.32.

Table S1. List of compounds from AQVN group values 2.55 with centroid distance from the learning set

ZINC20762773	1.0444
ZINC03880726	1.2448
ZINC00756618	1.3382
ZINC04081390	1.5085
ZINC19321394	1.6387
ZINC15121960	1.6589
ZINC72326056	1.8159
ZINC00322074	1.8524
ZINC01280229	1.883
ZINC20501905	1.9277

Table S2. List of compounds from AQVN group values 2.72 with centroid distance from the learning set

ZINC18277049	0.8405
ZINC18277048	0.9327
ZINC08764971	1.0001
ZINC04061177	1.2124
ZINC19323606	1.2864
ZINC08764993	1.3484
ZINC72324535	1.4092
ZINC12892770	1.5065
ZINC02543436	1.6168
ZINC03157475	1.6918

Table S3. List of compounds from AQVN group values 2.99 with centroid distance the from learning set

ZINC02115922	0.7866
ZINC31808017	0.8591
ZINC02122787	0.9713
ZINC02115924	0.97
ZINC02122785	0.9973
ZINC02149393	1.0433
ZINC00077345	1.0878
ZINC12296831	1.3264
ZINC00526223	1.3707
ZINC12874403	1.4146

Table S4. 5HT6 compounds learning set from ChEMBL database

CHEMBL3329438
CHEMBL3692995
CHEMBL1084794
CHEMBL398034
CHEMBL1085462
CHEMBL1086326
CHEMBL3692882
CHEMBL1083654
CHEMBL1085120
CHEMBL1086113
CHEMBL368209
CHEMBL1085658
CHEMBL24474
CHEMBL1086252
CHEMBL3692990
CHEMBL3692993
CHEMBL3692968
CHEMBL3696966
CHEMBL1082763
CHEMBL3692867
CHEMBL365569
CHEMBL1085037
CHEMBL1084711
CHEMBL1083886
CHEMBL363792
CHEMBL3692974
CHEMBL3692910
CHEMBL1922616
CHEMBL3692894
CHEMBL1085585
CHEMBL394690
CHEMBL1084336
CHEMBL1085617
CHEMBL1086079
CHEMBL1086323
CHEMBL3692988
CHEMBL2413990
CHEMBL3692989
CHEMBL1083390
CHEMBL3692997
CHEMBL3692863
CHEMBL1668590
CHEMBL3692975
CHEMBL3692971
CHEMBL1084603
CHEMBL1083781
CHEMBL1082508
CHEMBL373322
CHEMBL372879
CHEMBL2413982
CHEMBL3692973
CHEMBL46187
CHEMBL3329430
CHEMBL3329451
CHEMBL3692864
CHEMBL3329442
CHEMBL175835
CHEMBL1668584
CHEMBL1083075
CHEMBL1922632
CHEMBL1922615
CHEMBL3260311
CHEMBL3696953
CHEMBL3692866
CHEMBL3696956
CHEMBL3692996
CHEMBL1668586
CHEMBL1642864
CHEMBL1201756
CHEMBL3692901
CHEMBL196410
CHEMBL1086324
CHEMBL1085038
CHEMBL1084604
CHEMBL1082762
CHEMBL3669650
CHEMBL3692942
CHEMBL1668564
CHEMBL3692992
CHEMBL1950775
CHEMBL3693000
CHEMBL1950776
CHEMBL3692994
CHEMBL1668565
CHEMBL3669657
CHEMBL353552
CHEMBL179926
CHEMBL565723
CHEMBL3696954
CHEMBL96745
CHEMBL194307
CHEMBL3329453
CHEMBL609994
CHEMBL187865
CHEMBL193400
CHEMBL3329452
CHEMBL1922614
CHEMBL3669661
CHEMBL3669687
CHEMBL414628
